# Acylsugar amount and fatty acid profile differentially suppress oviposition by western flower thrips, *Frankliniella occidentalis*, on tomato and interspecific hybrid flowers

**DOI:** 10.1371/journal.pone.0201583

**Published:** 2018-07-31

**Authors:** Sulley Ben-Mahmoud, John R. Smeda, Thomas M. Chappell, Candice Stafford-Banks, Cassandre H. Kaplinsky, Taylor Anderson, Martha A. Mutschler, George G. Kennedy, Diane E. Ullman

**Affiliations:** 1 Department of Entomology and Nematology, University of California, Davis, California, United States of America; 2 Plant Breeding and Genetics Section, School of Integrative Plant Science, Cornell University, Ithaca, New York, United States of America; 3 Department of Plant Pathology and Microbiology, Agriculture and Life Sciences, Texas A&M University, College Station, Texas, United States of America; 4 Department of Entomology and Plant Pathology, North Carolina State University, Raleigh, North Carolina, United States of America; Chinese Academy of Agricultural Sciences Institute of Plant Protection, CHINA

## Abstract

Tomatoes (*Solanum lycopersicum* L.) have been bred to exude higher amounts or different types of the specialized plant metabolites, acylsugars, from type IV trichomes. Acylsugars are known to deter several herbivorous insect pests, including the western flower thrips (WFT), *Frankliniella occidentalis* (Pergande); however, all previous studies investigated the effect of acylsugars on leaves, or acylsugar extracts obtained from leaves. In spite of the WFT predilection for flowers, there is a gap in knowledge about flower defenses against thrips damage. This is especially important in light of their capacity to acquire and inoculate viruses in the genus *Orthotospovirus*, such as *Tomato spotted wilt orthotospovirus* (TSWV), in flowers. Therefore, we turned our attention to assessing thrips oviposition differences on flowers of 14 entries, including 8 interspecific hybrids, 5 tomato lines bred for specific acylsugar-related characteristics (type IV trichome densities, acylsugar amount, sugar moiety and fatty acid profile), and a fresh market tomato hybrid, Mt. Spring, which only produces trace amounts of acylsugars. Our results show that the density of the acylsugar droplet bearing type IV trichomes is greatest on sepals, relative to other flower structures, and accordingly, WFT avoids oviposition on sepals in favor of trichome-sparse petals. In concordance with past studies, acylsugar amount was the most important acylsugar-related characteristic suppressing WFT oviposition. Certain acylsugar fatty acids, specifically i-C5, i-C9 and i-C11, were also significantly associated with changes in WFT oviposition. These results support continued breeding efforts to increase acylsugar amounts and explore modifications of fatty acid profile and their roles in deterring thrips oviposition. The finding that acylsugar production occurs and reduces thrips oviposition in tomato flowers will be important in efforts to use acylsugar-mediated resistance to reduce incidence of orthotospoviruses such as TSWV in tomato by deterring virus transmission and development of thrips vector populations in the crop.

## Introduction

The western flower thrips (WFT), *Frankliniella occidentalis* (Pergande), has a host range exceeding 1200 plant species [1] and is a very significant pest of agricultural and horticultural crops worldwide [2, 3], inflicting damage to plants by sucking out contents of individual cells and transmitting viruses in the genus *Orthotospovirus* (family *Tospoviridae*) [1, 4–8]. Orthotospoviruses have an extensive host range of over 1,090 plant species [3, 7, 8] and the type member, *Tomato spotted wilt orthotospovirus* (TSWV), is estimated to cause annual worldwide losses of over $1 billion [9]. New orthotospoviruses and thrips vector species are rapidly emerging, with more than 25 tentative virus species and 14 thrips vector species now reported [8]. Western flower thrips earned the title *supervector* [10] due to their role as the primary vector of orthotospoviruses, high reproductive rates, polyphagous nature and resistance to most pesticides. The WFT is a major pest of tomato (*Solanum lycopersicon* L.), primarily as a vector of viruses (*Orthotospoviruses* and *Ilarviruses*) [11], but also by damaging fruit during feeding [6]. The WFT uses tomato as a temporary [12] or reproductive host [11, 13]; in the latter case, secondary spread of orthotospoviruses can be substantial. The importance of WFT as a global pest on tomatoes and other commercial crops has driven efforts to find sustainable and effective management strategies, including host plant resistance [14].

Acylsugar-mediated insect resistance has shown great promise for deterring multiple insect pests from tomato, including aphids, whiteflies and thrips [15–22]. Acylsugars are specialized plant metabolites produced by diverse species in the Solanaceae (*i*.*e*., genera *Nicotiana*, *Solanum*, *Petunia*, and *Datura*) [15–25], for which they are associated with insect resistance such as deterrence of a wide range of insect pests. Accessions of *Solanum pennellii* Correll, a wild relative of cultivated tomato, produce acylsugars that vary in amount and fatty acid profile among different accessions [26], and are secreted from type IV glandular trichomes that are present on all green parts of the plant [27, 28]. Acylsugars are sugar esters that are comprised of either a sucrose or glucose moiety with typically 3–4 fatty acid side chains that vary in orientation, length and location [22, 26, 27, 29–32]. The term fatty acid profile describes the types and relative amounts of the specific fatty acids present in a genotype’s acylsugars.

The Cornell University tomato breeding program crossed *S*. *pennellii* LA716, a wild relative of tomato that produces high amounts of acylsugars, and used subsequent backcross generations to derive the initial acylsugar producing tomato breeding lines CU97FL and CU071026 [16, 33]. As QTL affecting the amount of acylsugar production [16, 34–36] and/or differences in the sugar moiety and fatty acid profiles of acylsugars [35–38] were identified, CU071026 was used as a recurrent parent in further backcross breeding to add these QTL, thereby breeding a series of related tomato lines that produce acylsugars in different amounts or differing for fatty acid profiles [16, 38–40].

*In vitro* oviposition assays in which purified acylsugars from the tomato line CU071026 and four previously characterized *S*. *pennellii* accessions [26] were sprayed onto parafilm-covered sucrose substrates in equimolar amounts revealed differential functionality with regard to suppressing oviposition of the tobacco thrips (TT), *F*. *fusca* Hinds, WFT, and silverleaf whitefly (SLW), *Bemisia tabaci* (Gennadius) *MEAM1* (Middle East Asia Minor 1 biotype, former biotype B). Specifically, TT were sensitive to all acylsugars tested, while WFT and SLW were more sensitive to the acylsugars collected and purified from *S*. *pennellii* accessions than those collected and purified from the benchmark acylsugar accumulating tomato line CU071026 [22]. Acylsugars from the *S*. *pennellii* accessions differed from the acylsugars of CU071026, which produces nearly 100% acylsucrose, in that they accumulated almost exclusively acylglucose or a mix of acylsucrose and acylglucose. In addition, their fatty acid profiles differed. Synergism occurred when fractions from *S*. *pennellii* LA1376 acylsugars differing in sugar moiety and fatty acid profile were combined [22]. Notably, the sugars and esterified fatty acid side chains also varied among the purified acylsugars tested, suggesting that particular combinations of sugar moiety and/or fatty acid side chains may play a role in WFT sensitivity as measured by oviposition. Field trials using some of the new tomato lines and interspecific hybrids with differences in acylsugar amount, sugar moiety, and acylsugar fatty acid profile [38–40] also support the role of acylsugar amount and fatty acid profile in altering thrips densities (Kennedy et al. unpublished). Taken together, these data support the hypothesis that thrips species respond differentially to acylsugar amount and that the chemistry of the sugar moieties and fatty acid profiles may be intrinsic to deterring WFT oviposition.

True to its name, the WFT is often found in flowers [41, 42] although it is also known as a polyphagous omnivore that regularly feeds on all above ground plant tissues and opportunistically engages in predation [4, 41, 43]. Most investigations of insect responses to diverse acylsugars focused on acylsugars that were purified from leaves of *S*. *pennellii* accessions or of CU071026 [16, 22] and on *in planta* testing of CU071026 and newer tomatoes developed by the Cornell breeding program for differences in acylsugar amount, sugar moiety and fatty acid profile [16, 22, 33, 44]. In light of WFT predilection for flowers, we turned our attention to exploration of type IV trichome distribution, acylsugar amount and fatty acid profile in flowers, and evaluation of their impact on WFT oviposition responses. Specific understanding of thrips oviposition choices relative to acylsugar production and fatty acid profile in flowers is important because orthotospoviruses are efficiently acquired by thrips during larval stages, [8, 45] and adults become infective after the viruses replicate and invade the principal salivary glands [12, 13]. Consequently, continuity of the viral transmission cycle is dependent upon thrips laying eggs on plants that support thrips and virus development [14]. Due to this intimate relationship, oviposition preference not only determines offspring fitness, it dictates virus transmission [46]. Furthermore, WFT can inoculate TSWV to tomato flowers, causing infections and symptoms in the developing fruit [47]. For these reasons, thrips responses to flowers play an important role in the TSWV transmission cycle and flowers may either contribute to, or provide a potential escape from resistance mechanisms. Hence, we tested the hypothesis that type IV glandular trichomes and acylsugar production would occur on flowers, and that acylsugar amount and fatty acid profile would influence WFT oviposition on flower structures.

## Materials and methods

### Organisms

Western flower thrips (colony initiated from thrips collected in Hawaii, 1985) were reared on green bean pods (*Phaseolus vulgaris* L.) as described previously [48]. The entries used included interspecific hybrids, acylsugar breeding lines and hybrids from the Cornell breeding program, and a cultivated tomato control. Densities of type IV trichomes were measured on a subset of entries (see below), and chemistry of acylsugars produced, and thrips oviposition on flower structures with varying acylsugar amounts and fatty acid profiles were evaluated on all of the plant entries.

The commercial tomato hybrid “Mt. Spring” [49] was included as a negative control. Like all cultivated tomatoes, Mt Spring has simple trichomes that produce and accumulate low acylsugar amounts compared to those of acylsugar producing entries. Seeds of all other entries were produced at Cornell by Mutschler’s program. The Cornell acylsugar line, CU071026, has been the benchmark acylsugar positive control in experiments comparing acylsugar lines and interspecific hybrids for *ca*. 11 years [16, 22, 40]. To enhance continuity of comparisons, we have included this line as an acylsugar positive control. Two entries, ASX-1 and ASX-2, were acylsugar tomato hybrids created by crossing two acylsugar-producing tomato lines that differ for one or more introgressions for increased trichome density and acylsugar quantity [16]. Both hybrids, ASX-1 and ASX-2, share one parent that has the same *S*. *pennellii* introgressions as CU071026 plus the chromosome 6 QTL that increases trichome density and acylsugar amount [22]. Due to the differences in the second parents of the two hybrids, entry ASX-1 also has the TSWV resistance gene, *Sw-5*, and entry ASX-2 possesses the *Ty-3* and *Tm-2* genes for resistance to *Tomato yellow leaf curl virus* and *Tomato mosaic virus*, respectively. Notably, the large chromosome 9 introgression carrying *Tm-2* is also associated with an increase in acylsugar amount (Mutschler, unpublished). In contrast, the second parent for hybrid, ASX-2, had a modification in its chromosome 3 *S*. *pennellii* introgression that moderately lowers total acylsugar accumulation. Entries FA2/AS and FA8/AS each possess one additional introgression with QTL altering fatty acid profiles. FA2/AS has an acylsugar fatty acid profile characterized by an increase in extended branched chain fatty acids (i.e. i-C11 and i-C13), whereas FA8/AS has an acylsugar fatty acid profile with a significant increase in i-C4 fatty acids [38–40].

The remaining nine plant entries used were interspecific F1 hybrids created by crossing a tomato line as the female parent with a *S*. *pennellii* accession as the male parent. These interspecific hybrids were used to expand the differences represented in acylsugar amount and fatty acid profile represented among the entries used. The interspecific hybrids ISX-1, ISX-2, ISX-3, and ISX-4 were created by crossing the tomato line NC1CELBR (female parent) with one of four *S*. *pennellii* accessions as a male parent. Three additional interspecific F1 hybrids (ISX-6, ISX-7, and ISX-8) were produced by crossing the acylsugar tomato lines CU071026 (female parent) with three of the same *S*. *pennellii* accessions as a male parent. The four S. *pennellii* accessions used as male parents in these hybrids were selected based on results from previous studies of thrips responses to purified acylsugars applied to parafilm substrate [22]. Those investigations indicated that acylsugars with a glucose moiety might differentially decrease oviposition by WFT. Hence, a final interspecific hybrid (ISX-9) was created to raise the percentage of acylglucoses produced. This was done by crossing a tomato line carrying the QTL AGLU3 and AGLU11 (Mutschler, unpublished), which are two of the three *S*. *pennellii* QTL needed for acylglucose production [36], as the female parent with *S*. *pennellii* LA716 as a male parent.

Seeds were sown and plants maintained in the greenhouse until they were ready for transplanting. Seedlings of all plant entries used in our experiments were transplanted directly to the field on the University of California Davis (UCD) campus during the 2014 summer season (May-September) or transplanted to pots maintained in a UCD campus lathe house. At the time of flower collection, CU071026, ASX-2 and ISX-9 were no longer producing flowers in the field, but were still producing flowers in the lathe house. Consequently, for these lines/interspecific hybrid, flower collections were made from these plants in the lathe house.

Seedlings were planted in the field in a randomized complete block design with 5 replicates per entry consisting of 20 ft plots with 5 plants/plot spaced 5 ft apart. Rows were 5 ft apart. In the lathe house, a similar design was followed. Plants were grown in 1-gallon pots, with each pot placed equidistant from adjacent pots such that leaves did not touch each other.

### Trichome density on flower structures at four flower stages, and western flower thrips oviposition experiments

Type IV trichome density and thrips oviposition counts (adapted from a technique for counting leafhopper eggs) were measured using previously described methods [39, 50] on tomato flowers at four stages of development. Stage one included flowers in an early stage of development (small buds with fully closed sepals and enclosed petals). Stage two flowers had partially opened sepals, revealing developing petals still fully enclosing reproductive organs. Stage three flowers had fully expanded sepals and prominent petals that were beginning to open and change color, revealing some of the reproductive organs. Stage four flowers were fully developed with sepals and petals fully open exposing reproductive organs.

For WFT oviposition experiments, flowers, where available and representative of stages 1–4, were collected from all mature tomato plants in each plot in the field and lathe house. To ensure that acylsugars and trichomes were not damaged during transit, flower peduncles were secured in 1% agar gel to hold flowers upright. Due to the labor-intensive nature of determining the number and distribution of type IV trichomes on flower structures, a subset of representative entries were selected for this study: CU071026 (Cornell acylsugar benchmark line), Mt. Spring (fresh market tomato hybrid), ASX-2 (acylsugar tomato hybrid) and ISX-9 (interspecific tomato hybrid). All structures (petal, sepal, stamen, and pistil) were removed from the base of the flower. Depending on flower stage and entry, individual petals and stamens occasionally required separation before removal from the base. Tissues were mounted on a microscope slide using double-sided tape with either the abaxial or adaxial side facing upward. Using a 10 x 10 grid micrometer in a Nikon SMZ-U Zoom 1:10 (Melville, NY) dissecting scope, the number of type IV trichomes present within 0.39 mm^2^ were counted from the proximal, middle and distal areas of each tissue type and side. Counts of trichomes on pistils and stamens were so low (most flowers had zero trichomes on pistils or stamens) that they were excluded from our analysis. Type IV trichome numbers were converted to type IV trichomes/mm^2^ for analysis and presentation.

### Flower acylsugar chemistry

Amounts of accumulated acylsugars for all entries were measured on 9–10 week old plants similar to the method discussed in Leckie et al. [16]. Four samples (flowers) from each of 8 plants per entry were collected for chemical analysis. Each set of flowers was placed in a wide mouth plastic scintillation vial (Research Products International, IL, USA) and completely dried. Fully dried flowers were rinsed with 3 ml of methanol (the assay uses 100 μl of each rinsate). Flowers were re-dried after rinsing and weighed, so that acylsugar amount could be expressed per weight dried flower tissue.

Variation for the acylsugar fatty acid profile of each entry was ascertained by re-suspending dried acylsugars rinsed from sampled flowers with 1 ml of methanol containing methyl heptanoate (30 mg L^-1^) as an internal standard, and then using transmethylation/GC-MS analysis, as described in Leckie et al. [38]. Peak areas of the resulting chromatograms were calculated using Varian MS Workstation Version 6.9.1 (Agilent Technologies, Santa Clara, CA, USA) and amounts of respective fatty acids were determined through comparison with levels of the internal standard to generate relative proportions of each fatty acid. The cultivated tomato, Mt. Spring, did not produce sufficient acylsugars to allow reliable evaluation of fatty acid profile.

### Comparison of western flower thrips oviposition on flower stages, structures, and among tomato and interspecific hybrids

We were interested in specific thrips oviposition responses to different stages and structures of flowers of individual tomato entries; therefore, non-choice oviposition experiments were designed and executed. Thrips oviposition on tomato flowers from each entry was measured on petals, stamens, sepals and pistils of flowers at the four stages of development (described previously) by exposing individual flower blossoms to 10 adult female WFT/flower for 24 h. The cultivated fresh market tomato hybrid, Mt. Spring, which only produces trace acylsugars was included as a negative control. The benchmark acylsugar line, CU071026, was included as a positive control. The remaining entries provided the opportunity to study the associations of varying acylsugar amount and fatty acid profile on thrips oviposition. Depending upon flower availability, each replicate included 3–7 flowers for each of the four flower stages for each entry, with each flower tested in an individual oviposition arena designed according to similar arenas described in Stafford et al. [51]. Each oviposition arena included an individual flower standing upright in a plastic vial with its peduncle embedded in 1% agar gel within the vial cap. Ten adult female WFT, 2 to 6 days post adult eclosion, were placed in each oviposition arena and held under a constant light source for 24 h at 25 °C. After removing thrips, flowers were individually immersed in a staining solution (McBride’s solution, prepared in the laboratory as follows—0.2% acid fuchsin in 1:1 ethanol: glacial acetic acid), and shaken on a Titer plate shaker (model 4625, Lab-line instruments, Melrose Park, IL, USA) at a low speed for 24 h. Flowers were then transferred to clean vials and soaked in a de-staining solution (prepared in the laboratory as follows—1:1:1 lactic acid: glycerol: water) on a Titer plate shaker at a low speed for 3 h, then moved to an incubator at 80 °C for 24 h. Following this treatment, thrips eggs, which are partially or fully embedded in plant tissues, were stained red and were readily visible under low magnification. Flowers were dissected under a microscope (Nikon, model SMZ-U, Melville, NY, USA) and eggs were counted in the petals, sepals, stamen, and pistils. Oviposition experiments were replicated three times.

### Statistics

Variation in trichome density was analyzed using the GLM procedure of the SAS system version 9.4 [52], fitting a model that included flower structure (petal or sepal) and entry name, and their two-way interaction, as independent variables. Least-squares means were estimated and t-tests for mean difference were conducted to identify differences between groups.

Variation in oviposition quantity was analyzed using the GLM procedure of the SAS system version 9.4, fitting a model that included: flower stage (stage 1 through 4), acylsugar amount (an average flower-wise amount across replicates for the given entry, in μmol g^-1^ flower weight), flower structure (petal, stamen, sepal or pistil), plant source (a binary classification indicating whether the plant material was initially sourced from the field or the lathe house), and the two-way interaction between acylsugar amount and flower structure. Oviposition was analyzed as a lognormally-distributed response variable. Again, least-squares means were estimated and t-tests for mean difference were conducted.

To study the role of fatty acid profile in affecting oviposition, variable selection using minimum-AICc (Akaike Information Criterion, corrected) was conducted, leading to a model fit using the independent variables listed in S1 Table. These include: acylsugar amount and amount of three specific acylsugar fatty acids (an average flower-wise amount across replicates for the given entry in μmol g^-1^ flower weight); these averages were used as independent variables in ANCOVA (Analysis of covariance). Variables were selected from the following pool: flower stage, acylsugar amount, entry, plant source, averages of flower-wise amounts across entries for each of the measured fatty acids, an average total flower-wise amount across entries of all fatty acids combined, an average total flower-wise amount across entries of all short chain (less than 8 carbons in length) fatty acids combined, an average total flower-wise amount across entries of all extended (8 or more carbons in length) fatty acids combined, an average total flower-wise amount across entries of all branched (iso or anteiso) fatty acids combined, an average total flower-wise amount across entries of all extended branched (iso or anteiso C8 or longer) fatty acids combined, and an average total flower-wise amount across entries of all straight chain fatty acids combined. Combinations of fatty acids were proposed to variable selection on the basis that types may have function in common, contributing additively.

The relative proportions of each fatty acid in the entries’ acylsugars were compared between entries by hierarchical clustering analysis (HCA) with a Pearson correlation using pairwise average-linkage clustering for both genotypes and acylsugars using the hierarchical clustering tools provided by GenePattern [53]. This analysis shows the relationship between entries based on the relative proportion of each individual fatty acid’s accumulation when compared to other entries, rather than comparing the total amount of fatty acid or the percentage of the fatty acid accumulating within an entry. It also provides information about which acylsugar fatty acids are more variable or more consistent across entries.

## Results

### Trichome density on flower structures at four flower stages

**Fig 1:**
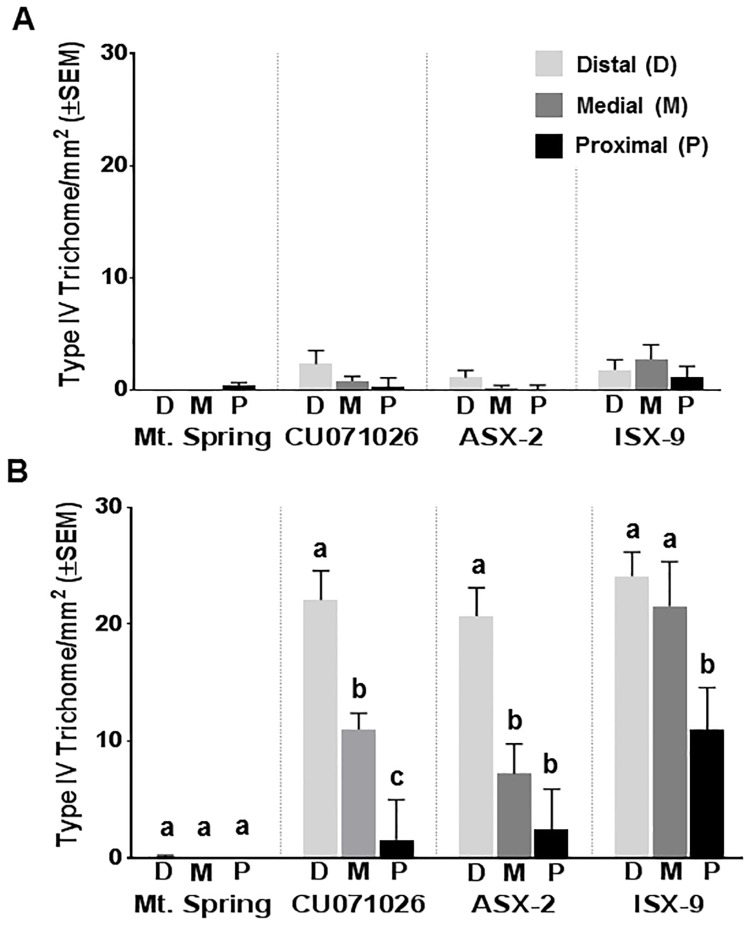
Mean type IV glandular trichome densities (type IV trichomes per square millimeter) on distal, medial and proximal sections of petals (A) and sepals (B) of four entries CU071026 (Cornell acylsugar benchmark line), Mt. Spring (fresh market tomato hybrid), ASX-2 (acylsugar tomato hybrid) and ISX-9 (interspecific tomato hybrid). Error bars represent the standard error of the mean (± SEM). Significantly greater numbers of type IV trichomes are located on sepals relative to petals and occur in decreasing densities from the distal to the proximal sections of the flower structures. The distal, medial and proximal portions of petals (Panel A in Fig 1), within an entry, are not significantly different, therefore, mean separations are not shown. Panel B in Fig 1 shows that among the lines with Type IV trichomes (CU071026 and ASX-2, the number of trichomes/mm2 was significantly higher on the distal portion of the sepal. With regards to the interspecific hybrid ISX-9, the number of Type IV trichomes/mm2 on the distal and medial portions of the sepal of ISX-9 were significantly higher than on the proximal portion of the sepal. Treatments with different letters above the bars are significantly different by pairwise t-tests at α = 0.05.

Type IV acylsugar-producing trichomes were found on all entries examined; however, the number of type IV trichomes found on the control, Mt. Spring, was negligible (Fig 1) hence, Mt. Spring was not included in further analysis of type IV trichomes/mm^2^. Significant differences were found in type IV trichomes/mm^2^ relative to entry, and, location of trichomes on and within flower structures (Table 1). Significantly greater numbers of type IV trichomes/mm^2^ were found on ISX-9 than on the acylsugar benchmark, CU071026, or ASX-2, while CU071026 and ASX-2 were not significantly different from one another (Table 1). Type IV trichomes were found on sepal, petal, and stamen tissues, but not on pistils. The numbers of trichomes on stamen tissues were so low that they were not included in our analysis. Significantly, more type IV trichomes/mm^2^ were located on sepals compared to petals (Fig 1). Furthermore, Type IV trichome densities were significantly higher on the distal portion of sepals, followed by medial and then proximal portions (Table 1, Fig 1). Distribution of type IV glandular trichomes was similar for adaxial vs. abaxial sides for all entries except ISX-9 which had more trichomes on the abaxial side of the tissues (data not shown).

**Table 1:** Comparison of Least squares (LS) means of type IV glandular trichome densities (type IV trichomes per square millimeter) across sepals of entries CU071026, ASX-2 and, ISX-9 and also across the distal, medial, and proximal portions of the sepals of the same entries, using lathe house grown plants.

Type of Comparison	Entry / Part of Sepal	Trichome LS Mean*	95% confidence interval
**Comparison among entries**	CU071026	11.54 a	8.78–14.30
	ASX-2	10.12 a	7.36–12.88
	ISX-9	18.86 b	16.10–21.62
**Comparison among different parts of the sepals**	Distal	22.29 c	19.53–25.05
	Medial	13.24 b	10.48–16.00
	Proximal	4.98 a	2.22–7.74

*LS means followed by the same letter are not significantly different at α = 0.05 by multiple t-test comparisons

### Flower acylsugar chemistry

**Fig 2:**
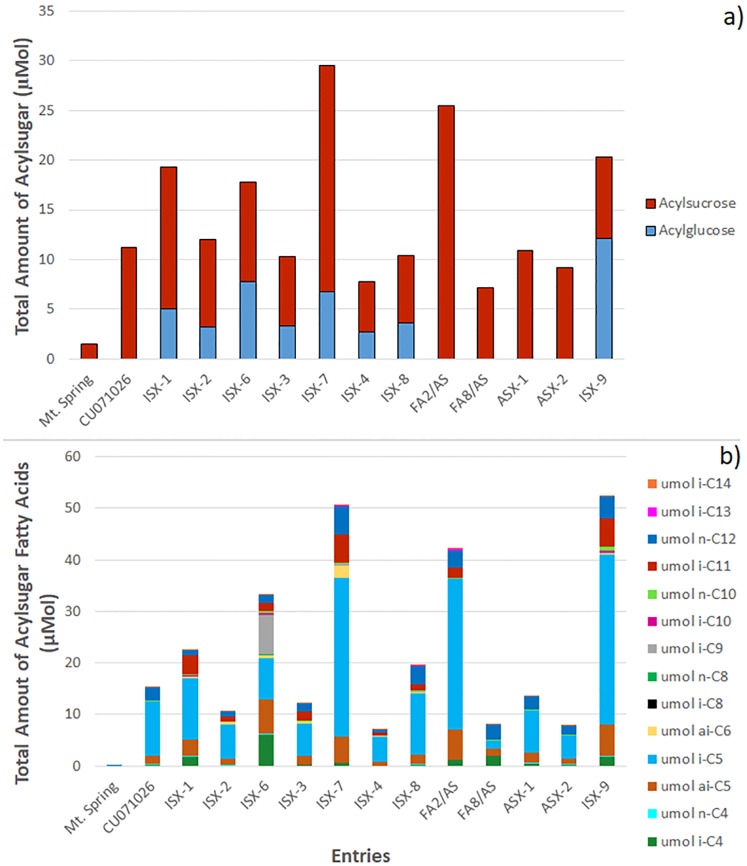
Stacked column charts depicting the total amount of acylsugars (acylsucrose and/or acylglucose) accumulated by each entry (a) and the total amount of the fatty acid components of the acylsugars accumulated by each entry (b). Amounts of acylsugars and acylsugar fatty acid components are measured in μMol per gram flower tissue. Acylsugar fatty acids constituting more than 0.25% of the total fatty acid profile of at least one entry were included in the analysis.

**Fig 3:**
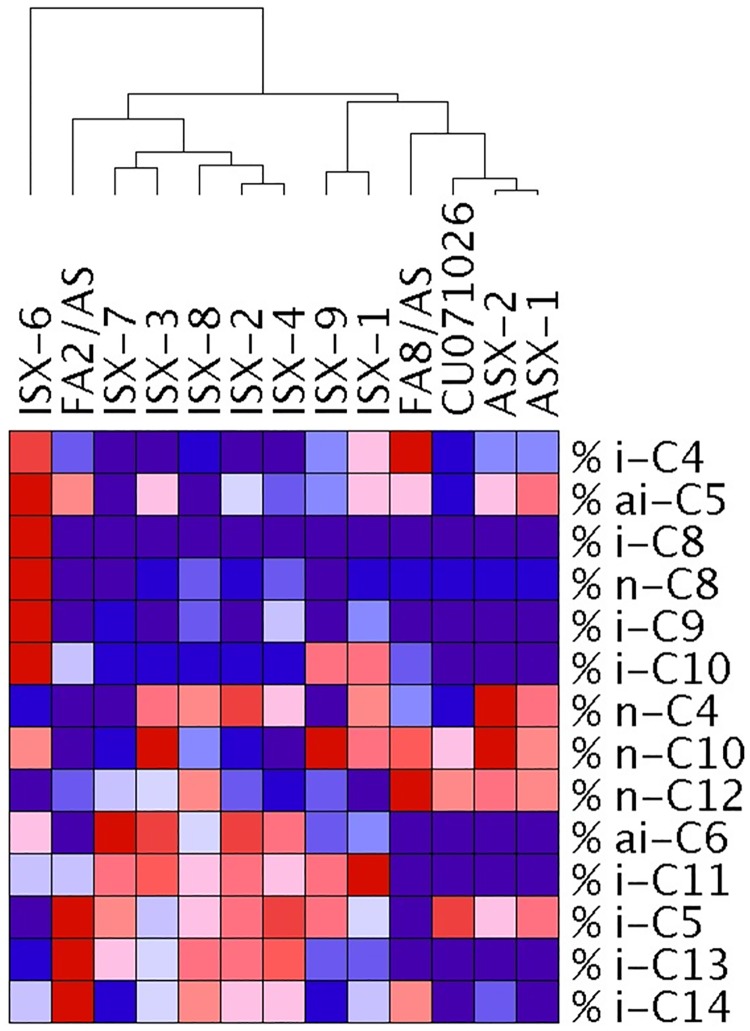
Hierarchical cluster analysis with Pearson correlation using a pairwise average-linkage clustering method, comparing the relative proportion of each acylsugar fatty acid by entry. Relative proportion of a fatty acid’s accumulation compared among the thirteen entries, excluding Mt. Spring, is shown. Color across a row indicates the relative proportion of an individual fatty acid as compared to all the other entries, but does not reveal the total amount of acylsugar fatty acid (see Panel B in Fig 2 for this information). Comparisons within an entry column are not relevant, because the colors do not infer total amounts or relative proportion of each fatty acid within an entry (see S2 Fig for this information). Dark red indicates that the percent accumulation of a fatty acid is the highest compared to the other entries in the row. Dark blue indicates that the percent accumulation of a fatty acid is the lowest (or absent) compared to the other entries in the row. Shade increase from dark blue through dark red indicates increasing percent accumulation of that particular fatty acid across the entries. Thus, the similarities and differences among entries for an individual fatty acid are shown by looking at the colors across a row.

The amounts of acylglucose and/or acylsucrose and the acylsugar fatty acid profiles varied substantially among entries (Fig 2a and 2b). The variation in the fatty acid profiles of the entries is also shown as proportions of the total acylsugar amount (S2 Fig). Given the diversity and complexity of the fatty acid profiles amongst entries, an understanding of individual fatty acids and relationships between entries is provided using hierarchical clustering analysis (HCA). This analysis examines the fatty acid profile of entries based on the relative proportion of an individual fatty acid’s accumulation across entries (Fig 3). Colors across a row indicate the relative proportion of an individual fatty acid as compared to all the other entries, but does not reveal information about the total amount of acylsugar fatty acids (see Fig 2b) or the proportion of a fatty acid relative to the total fatty acids within an entry (S1 Fig). Consequently, comparisons within an entry column are not relevant.

The relationships shown reveal that ISX-6 is on a branch separate from all the other entries, likely because of the relatively high proportion of the fatty acids ai-C5, i-C8, n-C8, i-C9 and i-C10 present when compared to all other entries (Fig 3). All the other entries cluster from a single branch likely due to their lack of i-C8, and zero, to relatively low proportions of n-C8, and i-C9. Among these entries, FA2/AS clusters in its own subgroup likely due to the relative proportions of fatty acids i-C5, i-C13 and i-C14. Notably, in this analysis, which compares all the entries tested together, FA2/AS, which is a tomato line, clusters most closely with the interspecific hybrids ISX-7, ISX-3, ISX-8, ISX-2 and ISX-4, rather than the other tomato lines, FA8/AS, CU071026, ASX-1, and ASX-2. Interspecific hybrids ISX-9 and ISX-1 clustered more closely with these four tomato entries than they did with the other interspecific hybrids. Therefore, these two entries formed an additional small cluster, although the fatty acids likely contributing to the relationship are not apparent.

To better understand the relationships between entries sharing a similar sugar moiety, entries were divided into two groups: tomato entries producing only acylsucrose or entries that are interspecific hybrids producing a mixture of acylsucroses and acylglucoses. The first group (Panel A in S1 Fig) consisted of tomato entries accumulating exclusively acylsucroses (FA2/AS, FA8/AS, CU071026, ASX-1 and ASX-2). The second group (Panel B in S1 Fig) of entries were interspecific hybrids that accumulated mixtures of acylsucroses and acylglucoses. CU071026 was included in both sets for comparison as the acylsugar benchmark control. Among entries accumulating exclusively acylsucroses (Panel A in S1 Fig), the FA2/AS entry was distinct from other entries likely because it accumulates i-C5, i-C9, i-C11, and i-C13 fatty acids in amounts greater than the other entries in this group. Notably, the relative proportions of i-C9, i-C11 and i-C13 fatty acids are absent or at trace amounts in FA8/AS, CU071026, ASX-1 and AX-2. FA8/AS was distinct from CU071026, ASX-1 and ASX-2 entries, likely due to the relatively high proportion of i-C4, ai-C6, n-C8, n-C12 and n-C10 found in this entry. CU071026 clustered with the closely related ASX-1 and AX-2 entries. Among entries that accumulated mixtures of acylsucroses and acylglucoses (Panel B in S2 Fig), ISX-6 is still on its own branch and was distinct, likely due to the relative proportion of ai-C5, i-C4, i-C10, i-C8, n-C8, and i-C9 fatty acids. The remaining interspecific hybrids separated into two major groups similar to those found in Fig 3 (ISX-9 and ISX-1 vs ISX-2, ISX-3, ISX-4, ISX-7 and ISX-8). Entries ISX-1 and ISX-9 cluster separately likely due to the relative increase in the proportion of i-C4 and i-C10 and decrease in ai-C6 fatty acids.

### Comparison of western flower thrips oviposition on different flower stages and structures

**Fig 4:**
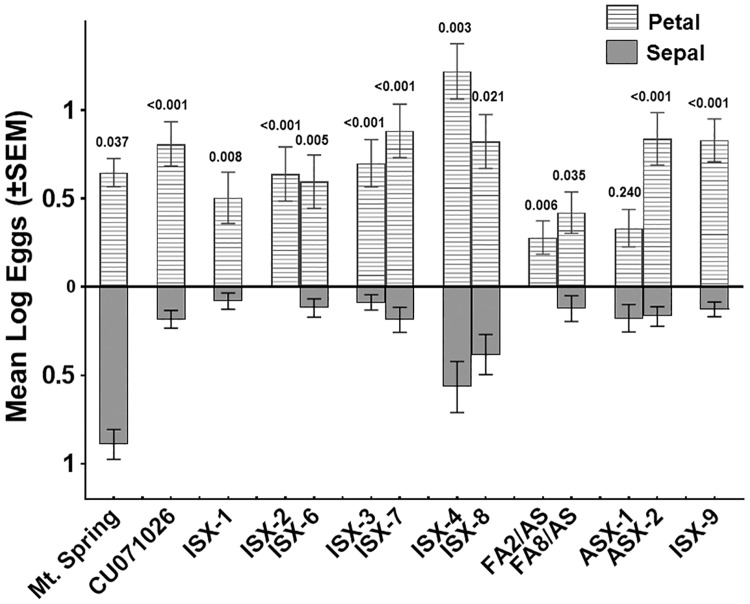
Comparison of *Frankliniella occidentalis* oviposition on petals (striped bars) and sepals (grey bars) of all entries. Significantly more eggs were laid on petals when compared to sepals on acylsugar-producing entries, except ASX-1, (*P* values resulting from entry petal by sepal t-test comparisons appear above error bars). This trend was reversed in the negative acylsugar control Mt. Spring, with thrips laying significantly more eggs on sepals compared to petals. The X-axis shows the identities of lines and interspecific hybrids (see Materials and methods for information on crosses). The Y-axis represents mean Log eggs with positive values in both upper and lower sections of the graph and error bars are the standard error of the mean (±SEM).

Flower stage and structure had a significant effect on thrips oviposition, explaining the greatest amount of variation in the model (Type III Sum of Squares = 176.189 and 34.546, respectively, *P* < 0.0001) (Table 2). Thrips oviposition increased significantly (α = 0.05) on mature flowers (older than stage 2). Thrips oviposition was lowest on flowers in the earliest stages of development (stages 1 and 2), which were not significantly different from one another (LS means of 0.118 and 0.184 respectively) and increased on flower stages 3 and 4. The greatest number of eggs was embedded in stage 4 flowers (LS mean, 0.843), about double and significantly different to stage 3 flowers (LS mean, 0.456). Thrips oviposition was greatest on petals, followed by stamen, sepals and then pistils (0.772^a^, 0.348^b^, 0.323^b^, and 0.158^c^ respective LS means—means sharing the same superscript letters are not significantly different at α = 0.05). Fig 4 shows that when oviposition on petals *vs*. sepals was compared entry-by-entry, thrips deposited more eggs on petals than sepals, with all but one pairwise difference being statistically significant. This ostensible difference (pertaining to ASX-1), and another marginally significant difference (pertaining to FA8/AS) occurred on entries on which very few eggs were oviposited. In contrast, on the cultivated tomato Mt. Spring, the trace acylsugar control, thrips laid significantly more eggs on sepals than petals (Fig 4).

**Table 2:** Analysis of variance of oviposition (egg count) by western flower thrips, *Frankliniella occidentalis* on flowers of thirteen acylsugar-producing entries and a cultivated tomato control producing only trace amounts of acylsugar^a^.

Source	DF	Sum of Squares	Mean Square	*F*	*P*
**Flower stage**^b^	3	176.189	58.730	152.95	< .0001
**Acylsugar amount**	1	12.550	12.550	32.69	< .0001
**Flower structure**^c^	3	34.546	11.515	29.99	< .0001
**Acylsugar amount x Flower structure**	3	27.227	9.076	23.64	< .0001
**Plant Source**^d^	1	8.192	8.192	21.33	< .0001

^**a**^Descriptions of entries can be found in Materials and methods

^**b**^**Flower Stage**: **Stage one**: small buds with fully closed sepals and enclosed petals. **Stage two**: flowers partially opened sepals, revealing developing petals still fully enclosing reproductive organs. **Stage three**: fully expanded sepals. Petals prominent, beginning to open and change color, revealing some of the reproductive organs. **Stage four**: flowers were fully developed with sepals and petals fully open exposing reproductive organs.

^**c**^**Flower structure**: sepals, petals, anthers, pistil

^**d**^**Plant Source**: Field vs lathe house

### Comparison of western flower thrips oviposition differences among tomato and interspecific hybrids

**Fig 5:**
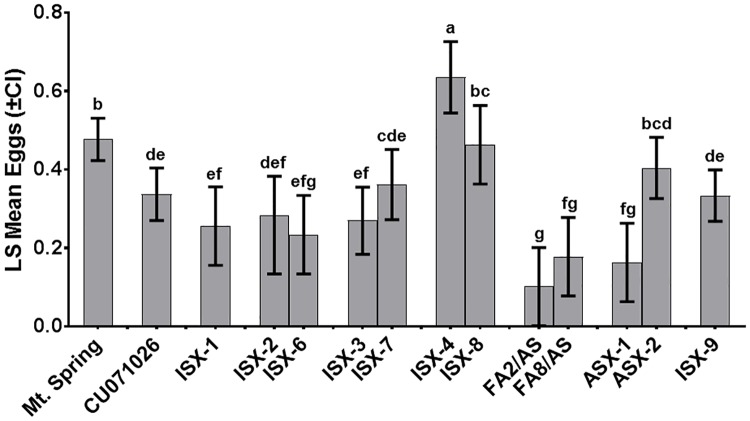
*Frankliniella occidentalis* oviposition on flowers of tomato, by entry. Least squares means of total egg count (across all structures and flower stages), which shows variation in aggregate oviposition associated with each of the entries. The unit scale is the natural logarithm of egg count. Pairwise t-tests were conducted to detect differences between entries’ means. Error bars include 95% confidence intervals. Entries sharing an above-bar letter are not different by pairwise t-tests at α = 0.05.

Fig 5 shows how the entries differ with regards to estimated eggs/flower at a 95% confidence limit. Notably, thrips oviposited significantly more eggs on the interspecific hybrid ISX-4 than its interspecific hybrid pair ISX-8, which shares the same *S*. *pennellii* parent, or Mt. Spring. Other interspecific hybrid pairs (ISX-2 vs ISX-6) and (ISX-3 vs ISX-7) significantly suppressed WFT oviposition relative to the Mt. Spring, but were not different from one another or from CU071026. The greatest reduction in thrips oviposition occurred in response to flowers of FA2/AS. Thrips oviposition on this entry was significantly lower than on Mt. Spring, on CU071026, and on all other entries tested, except ISX-6, FA8/AS, and on ASX-1, a hybrid with increased density of type IV glandular trichomes on leaflets. It is important to note that when oviposition on petals vs. sepals is considered, all acylsugar-producing entries except ASX-1 significantly suppressed thrips oviposition on sepals when compared to Mt. Spring (Fig 4).

#### Effect of acylsugar amount on western flower thrips oviposition

Whereas the general effect of acylsugar amount per flower on oviposition is small, the amount of acylsugar appreciably affects oviposition when considered for individual tissue types. Specifically, oviposition is significantly suppressed on sepals (as previously indicated), the flower structure having the highest density of acylsugar producing trichomes and thereby the greatest accumulation of acylsugars (Fig 1). This interpretation is supported by partial regression coefficients shown in S2 Table: indicating a significant effect of acylsugar amount for oviposition on sepals (*P* < 0.0001).

#### Effect of acylsugar fatty acids on western flower thrips oviposition

Variable selection by minimum-AICc, conducted to identify an optimal set of input variables for a model oviposition on sepals, chose acylsugar amount and quantities of the three acylsugar fatty acids i-C5, i-C9, and i-C11 to be associated with variation in oviposition. Together, these three acylsugar fatty acids plus acylsugar amount explained a similar amount of variation as other models incorporating “entry” as a variable. Acylsugar amount (estimate ± standard error = -0.4949 ± 0.0466) explained the most variation in this model (Type III Sum of Squares = 39.221, *P* < 0.0001) and further revealed the negative correlation between increasing acylsugar amount and suppression of thrips oviposition (t = -10.60). Although to a lesser degree than acylsugar amount, the three fatty acids, i-C5, i-C9, and i-C11 (respective estimate ± standard error are: 0.0236 ± 0.0061, 2.6031 ± 0.6310, and -0.1146 ± 0.0426), explained a significant amount of variation after accounting for acylsugar amount (Type III Sum of Squares: i-C5 = 5.227, *P* = 0.0001; i-C9 = 5.944, *P* < 0.0001; i-C11 = 2.524, *P* = 0.0074). Increasing amounts of i-C5 and i-C9 were associated with increased oviposition (t = 3.87 and 4.13 respectively), while increasing i-C11 amount was associated with suppression of thrips oviposition (t = -2.69).

## Discussion

While many studies have investigated type IV trichomes on leaves, our study is the first to show that type IV trichomes occur on parts of tomato flowers and produce acylsugars. Importantly, WFT oviposition on flowers was suppressed by increasing acylsugar amount, and at least three fatty acid components were associated with changes in WFT oviposition preferences, one of which was associated with suppression of oviposition. The importance of acylsugar amount and fatty acid profile in suppressing thrips oviposition on flowers is consistent with earlier work in which effects of purified acylsugars from leaves on WFT oviposition were shown to be concentration dependent and vary depending on acylsugar type [39]. The WFT is known for its preference for flowers [54, 55], typically ovipositing on virtually all flower structures across an extraordinarily large host range. When WFT accesses a flower, the insect can choose between flower structures as oviposition sites. Our results show that in a floral environment lacking significant production of type IV trichomes and their associated acylsugars, e.g. the cultivated tomato Mt. Spring, WFT oviposits eggs on several flower structures, including sepals, stamens and petals, with a significant preference for sepals (Fig 4). In contrast, among a subset of tested acylsugar-producing entries, type IV trichomes and associated acylsugars are concentrated on sepals (Fig 1), and in this situation, thrips were nearly uniformly deterred from ovipositing on sepals, while ovipositing preferentially on petals (Fig 4). Oviposition on petals occurred even when the insects were fully deterred from ovipositing on sepals, e.g. ISX-2 and FA2/AS (Fig 4). We interpret this to suggest that when WFT are seeking an oviposition substrate in a closed environment and they encounter a repellent surface they shift their oviposition efforts to a less repellent surface nearby if one is available. In our experiments, surfaces with fewer type IV trichomes and acylsugars, such as the petals and/or stamen of the same flower, were the alternatives available to the thrips. Hence, the stamen and petals of acylsugar producing entries may provide an “escape” from acylsugar-mediated resistance by supporting significant WFT oviposition. This may then also allow for inoculation of orthotospoviruses via stamen and petals. We suggest it seems likely that when thrips are not confined and they encounter a repellent surface, they may choose to leave rather than oviposit. Notably, one of the entries we tested, which displays a distinct fatty acid profile (FA2/AS), fully suppressed WFT oviposition on sepals and oviposition on petals was also the lowest among the entries tested (Fig 4). It is possible that WFT sensitivity to FA2/AS will be great enough that in an open environment the insect would likely choose to leave, thus reducing the role these flowers may play as a resistance escape and as an avenue for virus inoculation. Our results inform future experiments that will seek to elucidate the relative importance of acylsugars that fully repel thrips from an encountered flower. Given the importance of flowers in field epidemiology of orthotospoviruses, selection of tomatoes accumulating acylsugars that are repellent to thrips feeding and oviposition on flowers will benefit the outcomes of future breeding and disease management efforts.

We examined variation among acylsugars produced by the entries in this study in three ways: 1) relative amounts and proportions of acylsucrose versus acylglucose; 2) fatty acid profile reflected by the relative amounts and proportions of different fatty acids; and 3) the total amount of acylsugars accumulated. We did not detect any associations between the amounts and proportions of acylsucrose versus acylglucose and thrips oviposition; however, our findings strongly support the hypothesis that variation in acylsugar amount affects insect response, and that particular acylsugar fatty acids, i-C5, i-C9 and i-C11, may interact to modulate effectiveness of acylsugar exudate in deterring insects. Our experiments do not measure a cause and effect relationship between particular fatty acids and thrips oviposition; however, our findings support the hypothesis that when acylsugar amount is accounted for, other aspects of acylsugar chemistry, such as fatty acid profile, interact with acylsugar amount and play a role in altering thrips oviposition responses. Future work should examine the role of sugar moiety and the interactions between sugar moiety and fatty acid profile in modulating thrips oviposition responses. The Cornell program is developing new modified-acylsugar tomato lines to address these interactions and refine our understanding of insect responses to varying acylsugars [39, 40, 44]. Notably, we have already shown that a tomato bred to produce almost exclusively acylglucoses was associated with reductions in WFT oviposition on leaves equivalent to the reductions on leaves of CU071026, but results in significantly lowered inoculation of TSWV by WFT compared to CU071026 [44]; this acylglucose producing tomato line has not yet been tested for oviposition in its flowers.

Our examination of flower acylsugar chemistry reveals the tremendous diversity found in the acylsugar-producing entries, including the varying amounts of acylsucrose vs. acylglucose and fatty acids (Fig 2), proportions of fatty acids (S2 Fig) and clustering based on the accumulations of individual fatty acids across these entries (Fig 3 and S1 Fig). The HCA analyses (Fig 3 and S1 Fig) are an important step towards understanding the relationships between these entries based on their fatty acids and will be useful as breeders and entomologists work towards dissecting integrated roles of acylsugar amounts, sugar moiety and fatty acid profiles in suppressing insect oviposition. Indeed, our findings show that there is not a single blend of acylsugar amount, sugar moiety and fatty acid profile that will serve as a "silver bullet”, but rather multiple blends of acylsugars may be effective. Given the propensity of WFT to develop resistance to control chemistries, this is an important finding that supports the use of acylsugars with diverse sugar moieties and fatty acid profiles to develop more sustainable host plant resistance against these important pests.

We were intrigued to find that WFT oviposition on flowers of interspecific hybrids ISX-4 and ISX-8, which share *S*. *pennellii* LA2560 as a female parent, was equal to or significantly greater than that observed on the negative control, Mt. Spring and all the other entries we studied (Fig 5). Significantly, fewer eggs were oviposited on sepals than on petals of these two entries (Fig 4), suggesting that acylsugars produced on sepals were still effective in suppressing thrips oviposition, although these two entries were among the least effective among the acylsugar-producing entries we tested. We do not know what attributes of the flowers of entries ISX-4 and ISX-8 contributed to increased overall thrips oviposition although accumulation of i-C5 and i-C9 (both associated with increased thrips oviposition) or flower morphology could play a role. Our findings with flowers differ from those found with purified acylsugars, in which purified extracts from *S*. *pennellii* LA2560 (a parent in ISX-4 and ISX-8) leaves had the highest efficacy in reducing WFT oviposition into treated parafilm membranes [19]. It is likely that this occurred because ISX-4 and ISX-8 are hybrids and the acylsugar accumulation is at or below the amount produced by CU071026. Furthermore, the fatty acid profiles of their acylsugars are markedly dissimilar to that of purified acylsugars from *S*. *pennellii* LA2560 [22].

Available vector management tools rarely control orthotospovirus spread or direct thrips damage, and plant resistance to thrips and orthotospoviruses has not been durable [56, 57]. These constraints, compounded by orthotospovirus diversity and emergence of new orthotospoviruses, and the need to feed growing human populations, require new knowledge-based technologies and integrated strategies for thrips and orthotospovirus management. Our findings add to growing support for integration of acylsugar-mediated resistance into sustainable management strategies against thrips and orthotospoviruses [15, 16, 22, 58, 59] and show that acylsugar-producing entries extend resistance to flowers, some with considerable efficacy, e.g. FA2/AS. This is of great practical importance because it supports efforts to stack acylsugar-mediated resistance with the *Sw-5* allele for resistance to TSWV as a means to preserve the durability of this resistance gene. The *Sw-5* gene is challenged by WFT’s capacity to inoculate flowers of plants carrying the *Sw-5* resistance allele [47]. The effect of increased acylsugar amount and particular fatty acid profiles on oviposition suppression we detected in flowers is consistent with similar findings from experiments involving leaves and purified acylsugars [22]. Taken together with promising preliminary data showing that acylsugar-mediated resistance may limit infection of plants with insect transmitted viruses [33, 44], this type of natural resistance has tremendous potential in sustainable crop protection of tomatoes, alone and in combination with resistance genes against plant viruses and other pathogens, e.g. *Sw-5*.

## Supporting information

S1 Fig Hierarchical cluster analysis with Pearson correlation using a pairwise average-linkage clustering method, indicating the predominant acylsugar fatty acids accumulated by each entry. Entries (excluding Mt Spring) were analyzed separately in two groups: (a) entries that accumulated exclusively acylsucrose acylsugars and (b) entries with mixtures of acylsucroses and acylglucoses. CU071026 is included in both groups for reference. Estimates for each entry are averages of 5–12 samples. Acylsugar fatty acid abundances within and across entries were normalized during analysis. Color across a row indicates the relative proportion (and not total amount) of a particular fatty acid, compared to all the other entries. Comparisons within an entry column are not relevant, because the colors do not infer total amounts or relative proportion of each fatty acid within an entry (see S2 Fig for this information). Dark red indicates that the percent accumulation of a fatty acid is the highest compared to the other entries in the row, whereas dark blue indicates that the percent accumulation of a fatty acid is the lowest (or absent) compared to the other entries in the row. Shade increase from dark blue, through dark red indicates increasing percent accumulation of that particular fatty acid across the entries. Thus, the similarities and differences among entries for an individual fatty acid are shown by looking at the colors across a row.
(TIF)

S2 Fig Stacked 100% column chart depicting the relative composition of acylsugar fatty acids accumulated by each entry. Acylsugar fatty acids that constitute more than 0.25% of the total fatty acid profile of at least one entry were included in the analysis.
(TIF)

S1 Table Analysis of variance of oviposition by western flower thrips, *F*. *occidentalis* on sepals of acylsugar-producing entries. (DOCX)

S2 Table Partial regression coefficients for parameters concerning interactions between acylsugar amount and flower structure. Differences in the relationship between acylsugar amount and oviposition for flower structures are indicated relative to that for stamens.
(DOCX)

S1 File Type IV glandular trichome counts on flower stage, structure and parts of four entries CU071026 (Cornell acylsugar benchmark line), Mt. Spring (fresh market tomato hybrid), ASX-2 (acylsugar tomato hybrid) and ISX-9 (interspecific tomato hybrid). (TXT)

S2 File Flower acylsugar chemistry data showing amounts of acylsugars and fatty acids on thirteen acylsugar-producing entries and a cultivated tomato control producing only trace amounts of acylsugar. (TXT)

S3 File Eggs deposited (oviposition) by western flower thrips, *Frankliniella occidentalis*, on flowers of thirteen acylsugar-producing entries and a cultivated tomato control producing only trace amounts of acylsugar. (TXT)
