# Supplementary figures and images for: Acylsugar amount and fatty acid profile differentially suppress oviposition by western flower thrips, Frankliniella occidentalis, on tomato and interspecific hybrid flowers
Source: PLoS One. 2018 Jul 31;13(7):e0201583. doi: 10.1371/journal.pone.0201583 (PMC6067722; doi:10.1371/journal.pone.0201583)

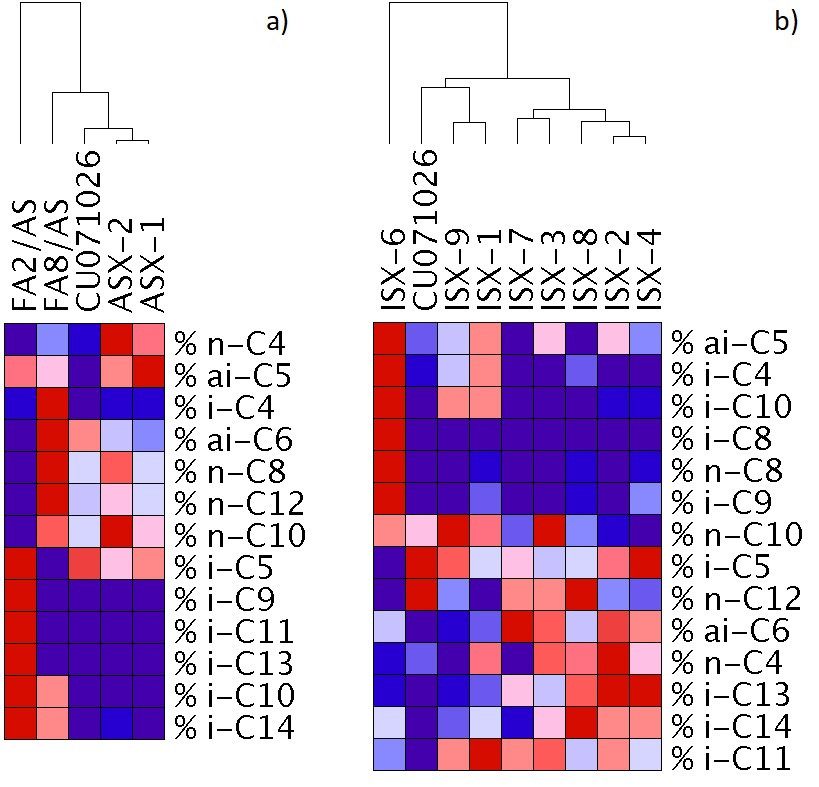

Supplement: S1 Fig — Entries (excluding Mt Spring) were analyzed separately in two groups: (a) entries that accumulated exclusively acylsucrose acylsugars and (b) entries with mixtures of acylsucroses and acylglucoses. CU071026 is included in both groups for reference. Estimates for each entry are averages of 5–12 samples. Acylsugar fatty acid abundances within and across entries were normalized during analysis. Color across a row indicates the relative proportion (and not total amount) of a particular fatty acid, compared to all the other entries. Comparisons within an entry column are not relevant, because the colors do not infer total amounts or relative proportion of each fatty acid within an entry (see S2 Fig for this information). Dark red indicates that the percent accumulation of a fatty acid is the highest compared to the other entries in the row, whereas dark blue indicates that the percent accumulation of a fatty acid is the lowest (or absent) compared to the other entries in the row. Shade increase from dark blue, through dark red indicates increasing percent accumulation of that particular fatty acid across the entries. Thus, the similarities and differences among entries for an individual fatty acid are shown by looking at the colors across a row. (TIF) [file pone.0201583.s001.tif]

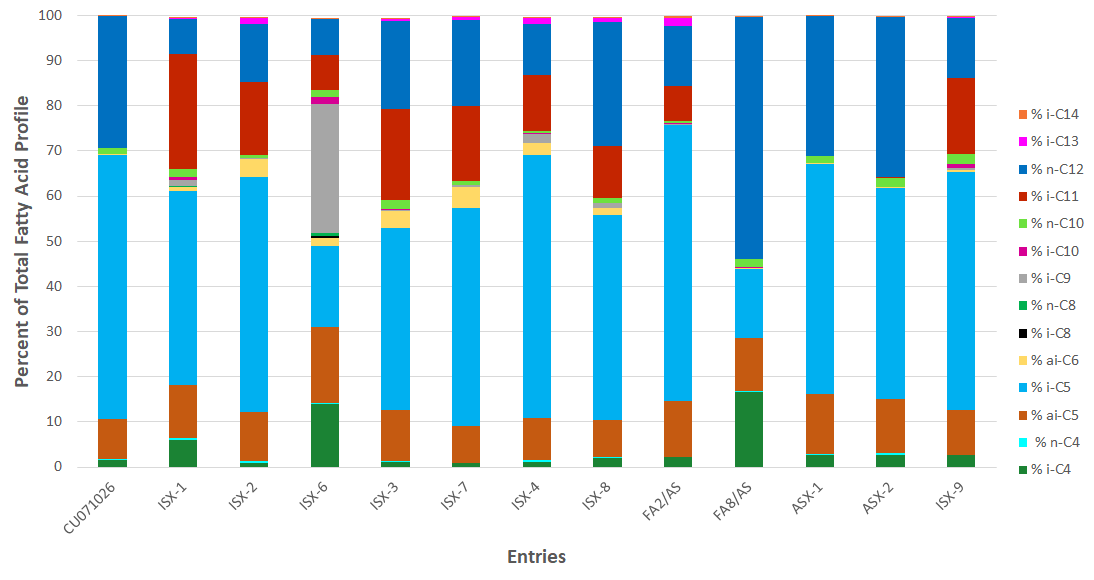

Supplement: S2 Fig — Acylsugar fatty acids that constitute more than 0.25% of the total fatty acid profile of at least one entry were included in the analysis. (TIF) [file pone.0201583.s002.tif]
