# Supplementary material for: Acylsugar amount and fatty acid profile differentially suppress oviposition by western flower thrips, Frankliniella occidentalis, on tomato and interspecific hybrid flowers
Source: PLoS One. 2018 Jul 31;13(7):e0201583. doi: 10.1371/journal.pone.0201583 (PMC6067722; doi:10.1371/journal.pone.0201583)
Supplement: S1 Table — (DOCX) [file pone.0201583.s003.docx]

**S1 Table.**

| **Source** | **DF** | **Sum of Squares** | **Mean Square** | ***F*** | ***P*** |
| --- | --- | --- | --- | --- | --- |
| **Acylsugar amount** | 1 | 39.221 | 39.221 | 112.28 | <0.0001 |
| **Average_i-C5** | 1 | 5.227 | 5.227 | 14.96 | 0.0001 |
| **Average_i-C9** | 1 | 5.944 | 5.944 | 17.02 | <0.0001 |
| **Average_i-C11** | 1 | 2.524 | 2.524 | 7.23 | 0.0074 |
