# Supplementary material for: Acylsugar amount and fatty acid profile differentially suppress oviposition by western flower thrips, Frankliniella occidentalis, on tomato and interspecific hybrid flowers
Source: PLoS One. 2018 Jul 31;13(7):e0201583. doi: 10.1371/journal.pone.0201583 (PMC6067722; doi:10.1371/journal.pone.0201583)
Supplement: S2 Table — Differences in the relationship between acylsugar amount and oviposition for flower structures are indicated relative to that for stamens. (DOCX) [file pone.0201583.s004.docx]

**S2 Table.**

| **Parameter** | **Estimate** | **Standard Error** | ***t*** | ***P*** |
| --- | --- | --- | --- | --- |
| **Acylsugar x Petal** | 0.0280 | 0.0429 | 0.65 | 0.5146 |
| **Acylsugar x Pistil** | 0.0709 | 0.0429 | 1.65 | 0.0989 |
| **Acylsugar x Sepal** | -0.2565 | 0.0429 | -5.97 | <.0001 |
| **Acylsugar x Stamen** | 0.0000 |  |  |  |
| **Acylsugar amount** | -0.0482 | 0.0304 | -1.58 | 0.1135 |
